# Defect Detection for Metal Shaft Surfaces Based on an Improved YOLOv5 Algorithm and Transfer Learning

**DOI:** 10.3390/s23073761

**Published:** 2023-04-05

**Authors:** Bi Li, Quanjie Gao

**Affiliations:** 1 Key Laboratory of Ministry of Education for Metallurgical Equipment and Control, Wuhan University of Science and Technology, Wuhan 430081, China; 2 Hubei Provincial Key Laboratory of Mechanical Transmission and Manufacturing Engineering, Wuhan University of Science and Technology, Wuhan 430081, China

**Keywords:** shaft defect detection, YOLOv5, attention mechanism, BiFPN, transfer learning

## Abstract

To address the problem of low efficiency for manual detection in the defect detection field for metal shafts, we propose a deep learning defect detection method based on the improved YOLOv5 algorithm. First, we add a Convolutional Block Attention Module (CBAM) mechanism layer to the last layer of the backbone network to improve the feature extraction capability. Second, the neck network introduces the Bi-directional Feature Pyramid Network (BiFPN) module to replace the original Path-Aggregation Network (PAN) structure and enhance the multi-scale feature fusion. Finally, we use transfer learning to pre-train the model and improve the generalization ability of the model. The experimental results show that the method achieves an average accuracy of 93.6% mAP and a detection speed of 16.7 FPS for defect detection on the dataset, which can identify metal shaft surface defects quickly and accurately, and is of reference significance for practical industrial applications.

## 1. Introduction

Shaft parts are widely used in the machinery manufacturing industry to support transmission parts, bear the load, and transmit torque, and their quality is essential in the production process [1]. However, in the actual production process, the surface of the shaft may produce various defects due to the influence of manufacturing equipment, process flow, and site environment. These defects can directly affect the performance and life of mechanical equipment. How to accurately and efficiently identify the surface defects of shafts and improve the production efficiency of shaft production lines is of great significance to enhancing the manufacturing industry. The traditional surface defect detection for metal shafts is mainly achieved by manual operation. Its drawbacks are apparent. Not only is it time-consuming and labor-intensive, it relies too much on the subjective judgment of workers, which can easily lead to low efficiency and poor reliability of defect detection for a long time, and the phenomenon of leakage and misdetection. Therefore, achieving fast, accurate, and efficient inspection of shaft surface defects is very important to improve product quality. With the advancement of machine vision and target detection technology, traditional machine vision-based inspection methods are widely used for defect detection and gradually replace manual inspection. Compared with traditional defect detection, machine vision inspection has the characteristics of fast detection speed and high efficiency. Yang et al. [2] proposed a method for surface defect detection using the smooth wavelet transform, which uses the Sobel operator to segment the image, and then uses indexing and non-linear filtering to remove the noise and extract the defect features from the object surface. Islam et al. [3] proposed a simple defect detection system by using boundary tracking and other image processing techniques. A simple defect detection system was proposed that solves the defect detection problem of capsules while using lower cost. Nikam et al. [4] proposed a simple method for fast localization and detection of printed circuit board defects, called the subtraction method, and used it to design a real-time image acquisition and inspection system for circuit boards. Although traditional machine vision-based inspection methods have achieved reliable results in many cases, they require our ability to extract representative feature information using specific pre-processing methods and specialized knowledge.

With the advent of the Industrial 5.0 era, production inspection automation has gradually become popular, and the technology of object surface defect detection based on deep learning has gradually matured, and the accuracy and efficiency of its recognition and detection can be comparable to that of humans. Deep learning detection is a surface defect detection algorithm based on the convolutional neural network, which can directly take the defect image as the input object of the network and automatically extract the image features, and finally output the defect recognition and classification results. Compared with the traditional machine vision detection methods, the deep learning-based detection method is highly accurate, fast, and adaptable. Park et al. [5] proposed a convolutional neural network-based surface defect recognition method for defects such as dirt and scratches that occur on metal parts during the production process, and the performance was better than manual detection. Masci et al. [6] applied a convolutional neural network to the steel defect classification task and achieved a better detection effect than the traditional SVM classifier. Liu et al. [7] investigated the application of Faster R-CNN in the defect location marking problem of textile defect detection, which can locate a fabric defect area more accurately and has good adaptability to various fabric images. Silvén et al. [8] proposed an unsupervised clustering-based defect detection and recognition method for sheet metal. Using a self-organizing mapping network to distinguish intact wood from defects, they achieved a low false detection rate and a low missed detection rate in an experiment with sheet metal color images. Li et al. [9] proposed a surface defect detection algorithm based on the deep learning MobileNet-SSD model and applied it to actual production. Yu et al. [10] proposed a lightweight network WM-PeleeNet based on PeleeNet modules for wafer defect detection, which reduces the complexity of the depth model by adjusting the structure of dense blocks. Compared with the original PeleeNet network, the parameters and flop of this network are significantly reduced.

Therefore, we apply the YOLOv5 algorithm to detect surface defects on metal shafts and improve on this method. To improve the feature extraction capability of the network, we add a Convolutional Block Attention Module (CBAM) mechanism layer to the last layer of the backbone network. To enhance the multi-scale feature fusion, the Path-Aggregation Network (PAN) structure in the original neck network is replaced with the Bi-directional Feature Pyramid Network (BiFPN) module of the bi-directional feature pyramid network. In addition, we also adopt a transfer learning approach to pre-train the model on the NEU-DET dataset to improve the model’s generalization ability. The main contributions of this paper are as follows:

1. We add a CBAM attention mechanism to improve the model’s expression effect and detection effect.
2. The original PANet feature fusion framework in the YOLOv5 neck network is replaced with the BiFPN module.
3. We use the transfer learning method to reduce the dependence of the training process on large samples.
4. All of these new features were tested and validated on the defect dataset of the metal shaft surface, and the conclusions confirmed the algorithm’s feasibility.

## 2. Related Work

### 2.1. Defect Detection Method Based on Traditional Machine Vision

The traditional machine vision detection method is based on the characteristics of the measured object to design the algorithm, which involves image processing algorithms that analyze the image texture, edges, skeleton, spectrum, and other feature attributes to discriminate and segment defects, thus achieving the extraction of target features. It includes structure class methods [11,12], threshold class methods [13,14,15], spectral class methods [16,17,18], and model class methods [19,20]. Liu et al. [21] detected pits and scratches on the surface of a bearing dust cover by using polar coordinate transformation and improved OTSU threshold. Shafarenko et al. [22] proposed a new color similarity measure and combined it with a watershed segmentation algorithm to achieve defect detection for random textures on surfaces. Medina et al. [23] proposed a rotation-invariant Gabor filter to detect defects in all directions for image processing.

However, traditional machine vision-based defect detection methods have many areas for improvement. Especially in the feature extraction part, different feature extractors usually need to be designed for different defect detection tasks, and the setting of critical parameters often relies on manual experience. Moreover, the traditional machine vision methods have limited shallow model expression capability, limited generalization capability for complex detection problems, are easily disturbed by environmental noise, and cannot meet the processing requirements of complex scenes.

### 2.2. Defect Detection Method Based on Deep Learning

Deep learning is a part of machine learning, but the difference between deep learning algorithms and machine vision detection methods is that the features are obtained differently. Deep learning has developed rapidly in the context of the significant data era, and its algorithms mainly rely on many data samples repeatedly and continuously training models. Deep learning abandons complex manually designed feature extraction algorithms to achieve end-to-end detection. Deep learning-based target detection models mainly include two major categories: one is a two-stage network based on detection frames and classifiers, represented by the Faster R-CNN [24] network; the other is a single-stage network based on regression, represented by the YOLO [25] and SSD [26] networks. Among them, the two-stage network first obtains the candidate frames that may contain defects before regression and classification, which usually has a higher accuracy rate of detection, but its high computational complexity of the candidate region extraction process makes the detection slower and cannot achieve real-time detection of targets; in comparison, the single-stage network directly uses the network to extract features to achieve defective target detection, which provides a vast improvement in the target detection speed and practicality. In comparison, the single-stage network directly uses the network to extract features to achieve defect target detection, which provides a vast improvement in target detection speed and is more practical and can be better applied in the actual production environment.

He et al. [27] proposed a deep learning-based defect detection method for steel plates. The method generates feature maps by applying a baseline convolutional neural network, merges features using a multi-layer feature fusion network, and generates regions of interest using a region suggestion network. Finally, the method achieves a mAP of 82.3 and a detection speed of 20 ft/s on a single GPU on the NEU-DET dataset. Evangelidis et al. [28] introduced a data-driven soft sensor model to replace high-end and low-cost sensors. The method was validated in printed circuit board manufacturing, reducing inspection time and achieving satisfactory results. Cha et al. [29] proposed an automatic detection of multiple types of damage based on Faster R-CNN, with an average detection accuracy of 87.8% for five types of defects, and combined with UAVs to achieve independent visual inspection of concrete and steel corrosion. Lv et al. [30] proposed an end-to-end defect detection network based on the Single Shot Multi-box Detector that can detect defects at different scales. It uses a complex harmful mining method to address data imbalance and data augmentation methods to enrich the training data. The method is robust and meets accuracy requirements for detecting metallic defects. Chen et al. [31] used an improved SSD network to detect surface defects on fasteners on contact network support devices and achieved an excellent defect detection rate and robustness. Li et al. [32] proposed a two-stage industrial defect detection framework based on an improved YOLOv5 and an optimized inception—resnetv2. The framework consists of two specific models for the localization and classification tasks. Moreover, experiments on several datasets, including natural industrial environments, achieved 83.3% and 91.0% mAP, respectively. Xiong et al. [33] introduced an improved deep-learning detection method for sheet defects based on YOLOv5. The method adds a novel attention module that fuses ECA-net and CBAM to improve the detection capability. The bottleneck CSP module is simplified to improve operation speed. Experimentally, the mapped value of the improved algorithm reaches 0.9578, which is more suitable than the original algorithm for detecting plate defects. Zheng et al. [34] proposed a YOLOv5 based on the squeeze excitation module. The model adds the SE module to the backbone of YOLOv5 and replaces the activation function of the cross-stage part of YOLOv5 with the ActivateOrNot activation function. The model achieves higher detection accuracy, generalization ability, and robustness in fabric defect detection.

Many research results show that deep learning methods are more accurate in target detection results and have more robust model generalization capabilities, overcoming the drawbacks of traditional digital image processing methods that can only detect specific types of defects. Deep learning techniques have their unique advantages in the detection field. However, they also have shortcomings that still need improvement, such as a training dataset that is too small; long, time-consuming model training; and excessive computational power consumption. To address these issues, we choose YOLOv5s as the initial model and improve it. Finally, we applied the improved YOLOv5s model to defect detection of metal shaft surfaces to improve the accuracy and rapidity of defect detection.

## 3. Proposed Method

### 3.1. Network Architecture

YOLOv5 contains four basic models, which are YOLOv5s, YOLOv5m, YOLOv5l, and YOLOv5x. Their network structures are the same, but the depth factor depth_multiple and width factor width_multiple of the network needs to be changed to adjust according to the task’s demand for detection accuracy and detection time. YOLOv5 first performs adaptive image scaling on the image to be detected. This is fed into the network to obtain an image with a pixel size of 640 × 640. The backbone network uses the cross-stage local network structure for feature extraction, and the neck network uses the network structure of FPN+PAN to combine the extracted features. Finally, the detection head predicts the location and class information for the target.

**Figure 1:**
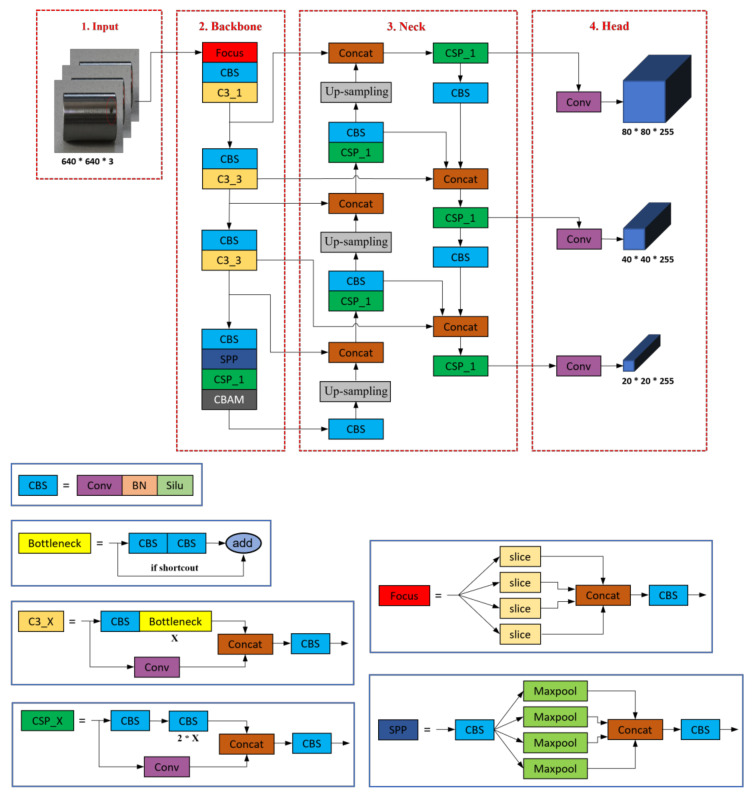
Improved YOLOv5 network structure.

Based on the YOLOv5s network structure, we made some improvements. First, for the backbone network, we add a CBAM attention mechanism layer in the last layer to enhance the network’s sensitivity to defect-related features and improve the overall detection accuracy of the network. Second, we introduce the BiFPN module for the neck network, which replaces the original PANet structure to achieve bi-directional feature information exchange and better multi-scale feature fusion. Figure 1 shows the structure of the improved YOLOv5 model.

### 3.2. Extended CBAM Mixed Attention Module

Based on the principle that humans selectively remember and recognize images when they observe objects, we add an attention mechanism to the YOLOv5 model. First, the model acquires the region that needs to be focused on. Then, the weight of that target region is increased while the weight of other useless information is decreased. We usually organize the algorithms in the attention mechanism into three primary attentions: spatial attention, channel attention, and mixed attention. Among them, the mixed attention calculates simultaneously the importance of channel and spatial attention.

**Figure 2:**
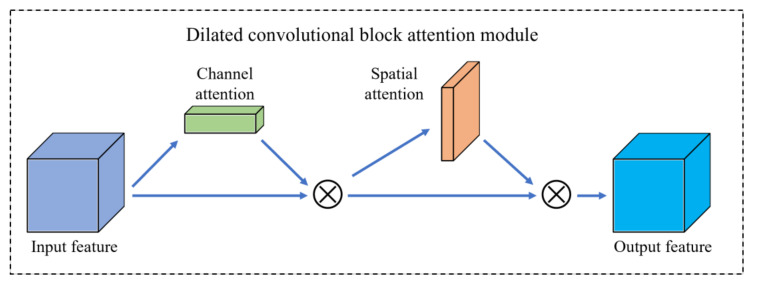
Schematic diagram of CBAM’s attention mechanism.

**Figure 3:**
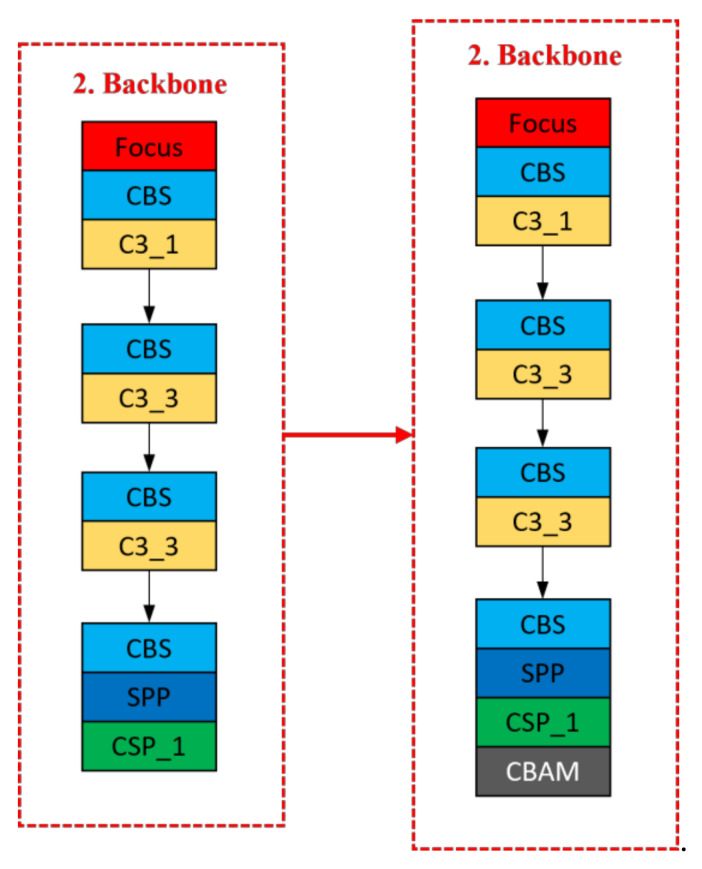
Backbone network structure with CBAM module added.

To extract the critical information required for the current task objective from the extensive feature information and to improve the efficiency and accuracy of image processing, this paper introduces the hybrid attention module CBAM [35]. This module is added to the last layer of the backbone network. As shown in Figure 2, the CBAM model has two main components: the channel attention module (CAM) and the spatial attention module (SAM). Figure 3 illustrates the improved backbone network structure.

Consider an intermediate feature graph $F\in R^{H\times W\times C}$ as input, where H, W, and C represent the feature map’s height, width, and number of channels, respectively. The CBAM model combines the CAM and SAM components by stacking them in parallel to compute simultaneously the channel and spatial attention maps. The resulting attention maps are then multiplied elementwise by element to selectively emphasize the feature information channels and suppress the irrelevant information channels to produce the final attention feature map. The following equations demonstrate the overall attention process:

(1) $$F^{\prime }=M_{c}\left(F\right)⊗F$$

(2) $$F^{\prime \prime }=M_{S}\left(F^{\prime }\right)⊗F^{\prime }$$

where ⊗ is by-elements multiplication, $M_{c}\left(F\right)\in R^{1\times 1\times C}$ is the characteristic of one-dimensional channel attention, $M_{S}\left(F\right)\in R^{H\times W\times 1}$ is the characteristic of two-dimensional spatial attention, $F^{\prime }$ is after channel attention to enhance the characteristics of the figure, and $F^{\prime \prime }$ is characteristic of after CBAM enhancement.

#### 3.2.1. Channel Attention Module

The CAM focuses on the interdependencies among feature channels. The CAM first performs mean-pooling and max-pooling on the input feature map *F*. Then, it outputs the channel attention weights after a multi-layer perceptron (*MLP*) containing two convolution layers. Immediately after that, the standard weight coefficients of each channel are calculated using the sigmoid activation function. Finally, each weight is weighted to the original channel, and the importance of different information on the number of channels of the initial feature map is reclassified. Equation (3) represents the process:

(3) $$M_{c}\left(F\right)=\sigma \left(MLP\left(AvgPool\left(F\right)\right)+MLP\left(MaxPool\left(F\right)\right)\right)=\sigma \left(w_{1}(w_{0}\left(F_{avg}^{c}\right))+w_{1}(w_{0}\left(F_{max}^{c}\right))\right)$$

where σ is the sigmoid activation function; *MLP* is a multi-layer perceptron; $AvgPool\left(F\right)$ and $MaxPool\left(F\right)$ are the mean-pooling features and max-pooling features, respectively; $w_{0}$ and $w_{1}$ are the shared weights values of *MLP*; and $F_{avg}^{c}$ and $F_{max}^{c}$ are the mean-pooling features and max-pooling features under one-dimensional mapping, respectively.

#### 3.2.2. Spatial Attention Module

The SAM, on the other hand, is concerned with the relationships between spatial locations in the feature map. The SAM first compresses the input feature map to a pixel size of 1 × 1 through a pooling layer. Then, the compressed feature map is compressed again in size by passing it through a 7 × 7 convolutional kernel and the ReLU activation function in turn. Immediately afterward, the compressed feature map is up-sampled using a convolutional kernel of size 1 × 1 to match the next input layer’s feature map size. Equation (4) represents the process:

(4) $$M_{s}\left(F\right)=\sigma \left(f^{7\times 7}\left(\left[AvgPool\left(F\right);MaxPool\left(F\right)\right]\right)\right)=\sigma \left(f^{7\times 7}\left(\left[F_{avg}^{s};F_{max}^{s}\right]\right)\right)$$

where σ is the sigmoid activation function; $f^{7\times 7}$ is a convolution operation with a filter size of 7 × 7; and $F_{avg}^{s}$ and $F_{max}^{s}$ are the mean-pooling features and max-pooling features under two-dimensional mapping.

### 3.3. BiFPN Characteristic Pyramid

**Figure 4:**
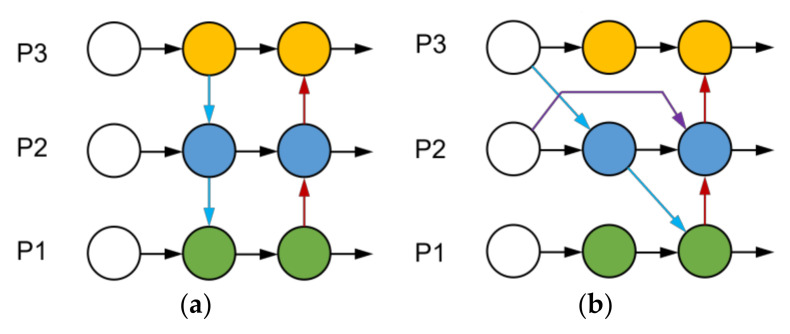
Structure diagram: (**a**) PANet (**b**) BiFPN. Circles represent each layer, black and colored lines represent the connection.

**Figure 5:**
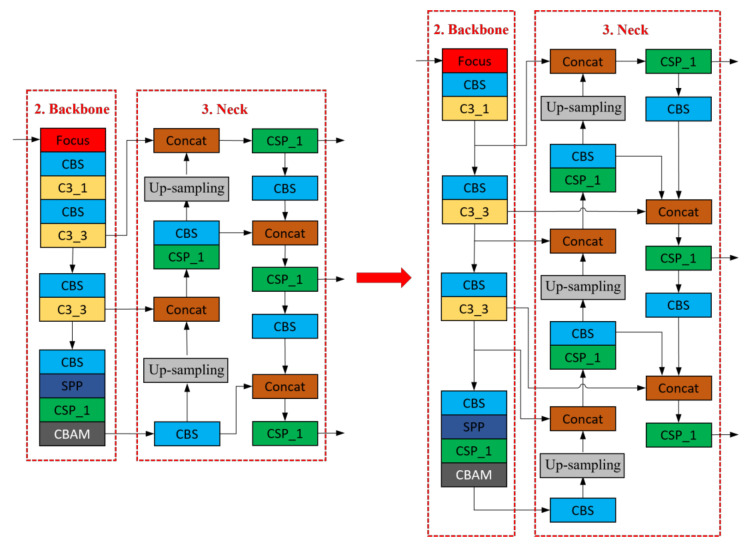
The network structure after BiFPN is adopted.

After the backbone network extracts the input image, it must be processed by the neck network and output to the detection part. The original YOLOv5s algorithm uses PANet as the neck network, and its structure is shown in Figure 4a. A simple summation operation achieves the information fusion between features and high-level features. However, in feature fusion, different input features contribute differently to the fused features, and the direct summation result cannot consider the importance of different features. For this reason, this paper adopts BiFPN [36] as the neck network, whose structure is shown in Figure 4b. BiFPN combines a top-down and bottom-up fusion of deep and shallow features in both directions and introduces learnable weights to learn the importance of different features, making the network give more attention to the feature mapping that contributes more to the output features. To describe the feature fusion of BiFPN at the second layer, for example, the following equation shows the computation of BiFPN:

(5) $$P_{2}^{td}=Conv\frac{\omega_{1}P_{2}^{in}+\omega_{2}Resize(P_{3}^{in})}{\omega_{1}+\omega_{2}+\theta }$$

(6) $$P_{2}^{out}=Conv\frac{\omega_{1}^{’}P_{2}^{in}+\omega_{2}^{’}P_{2}^{td}+\omega_{3}^{’}Resize(P_{1}^{out})}{\omega_{1}^{’}+\omega_{2}^{’}+\omega_{3}^{’}+\theta }$$

where $P_{2}^{td}$ is a top-down intermediate feature of the second layer; $P_{2}^{out}$ is a bottom-up output feature of the second layer; and ω is a learning weight parameter with a value between 0 and 1. *Resize* is an up-sampling or down-sampling operation, representing a minimal number, mainly to avoid numerical instability. Figure 5 illustrates the network structure before and after the adoption of BiFPN.

### 3.4. Transfer Learning

Deep learning models are often complex in structure. Therefore, if the training sample dataset is small, it can overfit the model and lead to its performance degradation. Transfer learning is a method to improve the training of a new model by using the parameters of a pre-trained model from another task. This approach is practical when there is a need for more data to avoid overfitting due to small datasets and to improve the training speed and performance of new models.

**Figure 6:**
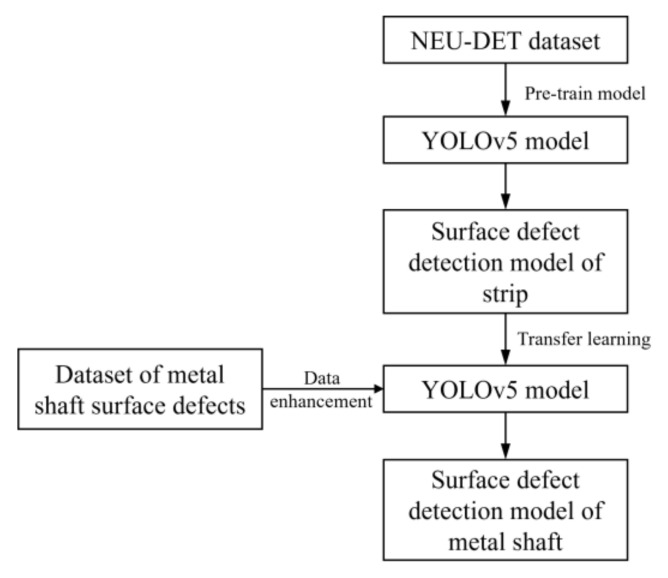
The transfer learning process described in this paper.

Yang et al. [37] used the CNN classical hierarchical model as an example to describe the application model of transfer learning. After deep extraction and integration of surface defect features of the metal shaft by the convolutional layer, the final deep feature information is input to the fully connected layer (FC) for classification. After using transfer learning, we usually discard the last FC layer and leave the other structures unchanged [38]. Then, new FC layers are trained and added to the structural layer according to three different cases. For this, we must compare the feature similarity between the defect datasets of NEU-DET and the metal shaft surface. In the first case, when the similarity between the old and new datasets is high and the difference in data volume is slight, we need to train the overall model and update the parameters after replacing the last FC layer. In the second case, when the similarity between the old and new datasets is high, and the data volume of the new dataset is much larger, we only need to replace the last FC layer and keep the other layer parameters unchanged. In the third case, when the similarity between the old and new datasets is low, and the data volume of the new dataset is much larger, we need to remove the last FC layer and do not need to add a new structure, initialize all parameters of the model, and then re-train it using the new dataset. Figure 6 shows the transfer learning method for training the detection model described in this paper.

## 4. Experiments Results and Analysis

### 4.1. Data Preparation

**Figure 7:**
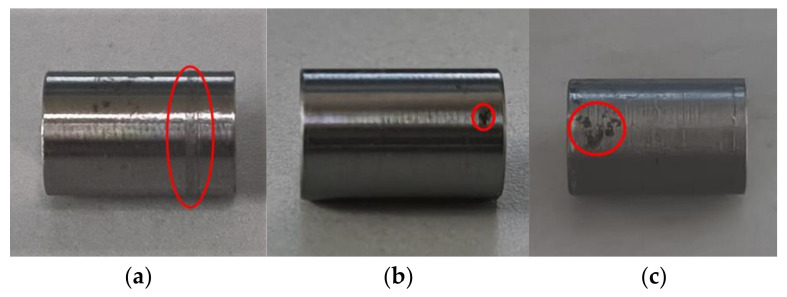
Three surface defect types on metal shafts: (**a**) scratches, (**b**) pitted, and (**c**) patches.

The dataset applied in this paper consists of two categories. One category is the NEU-DET surface defect dataset of strip steel provided by Northeastern University for pre-training the network model. This dataset includes surface defect images of six types of strip steel: cracks, inclusions, patches, pits, involvement, and scratches. Because the old and new datasets using transfer learning need high similarity, we select the defect images of three types of defects in the NEU-DET dataset: pits, patches, and scratches, to construct the dataset—300 images of each defect, a total of 900 images, for pre-training the network model. The other category is a self-made surface defect dataset of the metal shaft for re-training the network model. This data includes three types of defects: scratches, pitted and patches—100 images for each defect, 300 images in total. Figure 7 illustrates the three types of surface defects on metal shafts.

**Figure 8:**
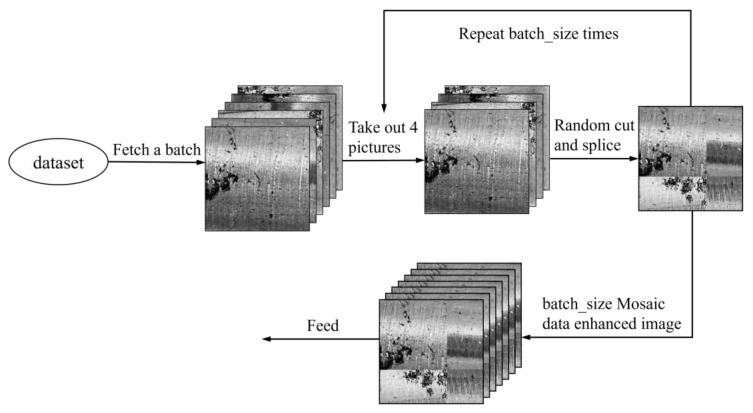
The mosaic data enhancement process.

Convolutional neural networks usually require many training samples to extract image features and perform detection and classification effectively. Since the homemade metal axis raw dataset is too small, the data need to be enhanced before the training starts to obtain better training results. In this paper, the dataset is initially expanded by standard methods such as mirror flip, brightness adjustment, and random cropping, and then expanded by mosaic data enhancement [39]. Mosaic data enhancement aims to cut and scale four images randomly, and then randomly arrange and stitch them into one image, which can enrich the dataset and increase the number of small samples. Figure 8 shows the defective images after mosaic data enhancement.

**Figure 9:**
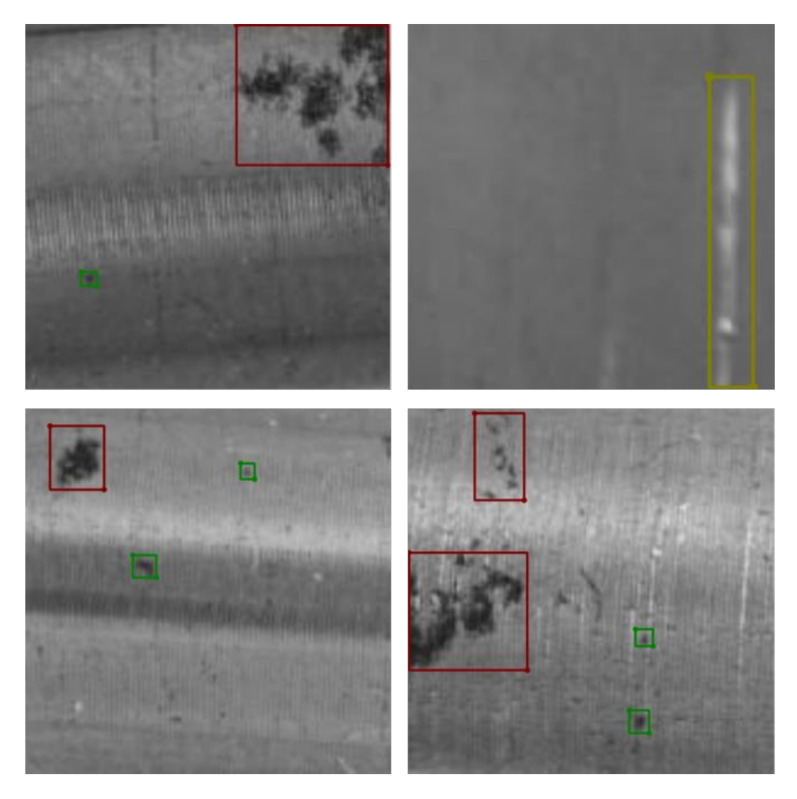
Annotated dataset. The colored boxes represent the drawn label boxes.

**Figure 10:**
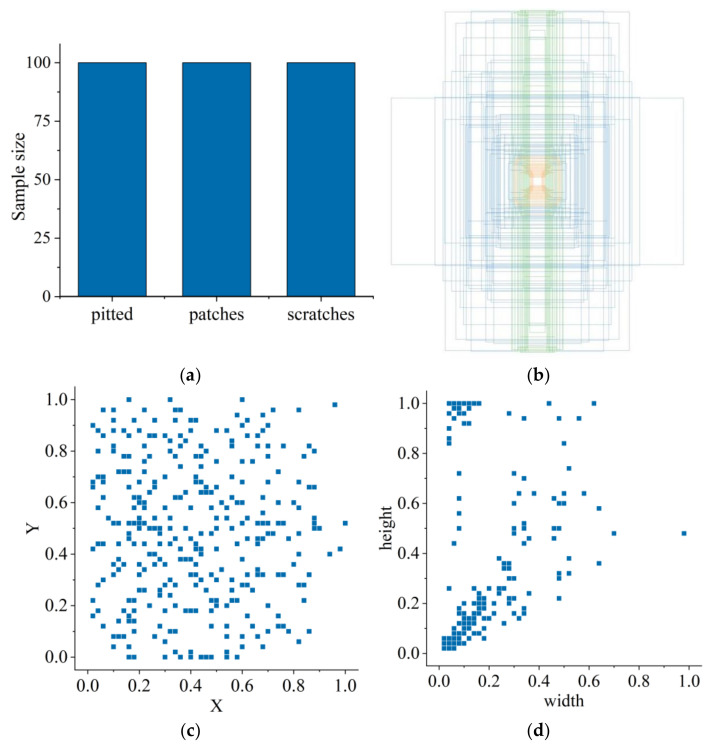
Category example of the metal shaft defect dataset: (**a**) Defect type and sample quantity. (**b**) Tag-box visualization. (**c**) The center point (x, y) of the label box distribution. (**d**) Label size distribution. The orange, blue and green boxes represent pitted, patches and scratches defects respectively.

Before training the YOLOv5 model, the images of metal shaft surface defects after data enhancement must be labeled and processed in advance. The method described in this paper uses the LabelImg labeling tool to label the image data manually. The labels of the labeled images are classified into three corresponding categories according to the three types of defects on the metal shaft surface: pitted, patches, and scratches. The labeling process automatically generates a file with a suffix ”json”, which contains information such as label categories and absolute coordinates of the actual frame. Then, the generated file with a ”json” suffix is converted to a file with a suffix ”.txt”, and the absolute coordinates of the actual frame are converted to relative coordinates. After completing the annotation of the surface defect image data of the metal shaft, the annotated data are randomly divided by code. We divide the training set, validation set, and test set according to the ratio of 8:1:1. Figure 9 shows the annotated dataset, where the colored boxes represent the drawn annotation boxes. Figure 10 shows an example of the defect dataset of the metal shaft surface.

### 4.2. Experiment and Parameter Determination

Our improved YOLOv5 model is implemented on the Windows 10 platform using the PyTorch framework. The Nvidia GeForce GTX 1050 graphics card is used for computing, and the video memory size is 4 G. The device uses Intel(R) Core (TM) i7-7700HQ CPU @ 2.80 GHz. The CUDA version is 10.1, the CUDNN version is 7.6, and the Python version is 3.7.

We divided this experiment into two parts. The first part is pre-training on the NEU-DET strip surface defect dataset, and the experiment sets the confidence threshold for the defect category to 0.5, the number of iterations to 300, the initial learning rate to 0.001, the batch size to 8, and the number of training categories to 3. After training, we obtain the optimal target detection model. The second part is to re-train the defect dataset of the metal shaft surface and experimentally set the confidence threshold of the defect category to 0.5, the number of iterations to 300, the initial learning rate to 0.001, the batch size to 8, and the number of training categories to 3. After training, we obtain the target detection model for metal shaft surface defects.

### 4.3. Network Evaluation

We used evaluation indexes such as precision (*P*), recall (*R*), mean average precision (*mAP*), and frames per second (FPS) detected per second to verify the identification performance for the improved YOLOv5 model applied to metal shaft surface defects.

Precision (*P*) refers to the positive sample predicted correctly in the forecast dataset. The number divides by the number of positive samples predicted by the model. Recall (*R*) refers to the number of correctly predicted positive examples in the forecast dataset divided by the positive actual number. *P* and *R* are calculated by using Equations (7) and (8).

(7) $$P=\frac{TP}{\left(TP+FP\right)}$$

(8) $$R=\frac{TP}{\left(TP+FN\right)}$$

where *TP* refers to positive samples that have been correctly allocated, and *FP* refers to positive models that have been incorrectly allocated. *FN* refers to negative examples that have been incorrectly allocated.

The *AP* value refers to the area of the *P*-*R* curve, and *mAP* is obtained by averaging the *AP* of all categories. *AP* and *mAP* are calculated by using Equations (9) and (10).

(9) $$AP=\int_{0}^{1}P\left(R\right)dR$$

(10) $$mAP=\frac{1}{\left|n\right|}\sum_{i=1}^{n}AP_{i}$$

where $AP_{i}$ represents the average accuracy of class *i* targets, and *n* represents the number of categories.

The *mAP*@0.5 value indicates the average sum of all *AP* when the intersection ratio (IOU) threshold is 0.5; *mAP*@0.5:0.95 represents the average sum of all *AP* that select the IOU threshold in an interval range (starting at 0.5 and increasing to 0.95 in steps of 0.05). The *mAP*@0.5 and *mAP*@0.5:0.95 values are calculated by using Equations (11) and (12).

(11) $$mAP@0.5=\frac{1}{n}\sum_{i=1}^{n}AP@0.5_{i}$$

(12) $$mAP@0.5:0.95=\frac{1}{10}\left(mAP@0.5+mAP@0.55+\ldots +mAP@0.95\right)$$

where *AP*@0.5 represents the average accuracy of class *n* target when the intersection ratio threshold is 0.5.

In addition, the number of frames per second (FPS) is used to evaluate the detection speed. Memory Usage (MB) is used to evaluate the calculation’s cost.

### 4.4. Performance Analysis of the Improved YOLOv5

**Figure 11:**
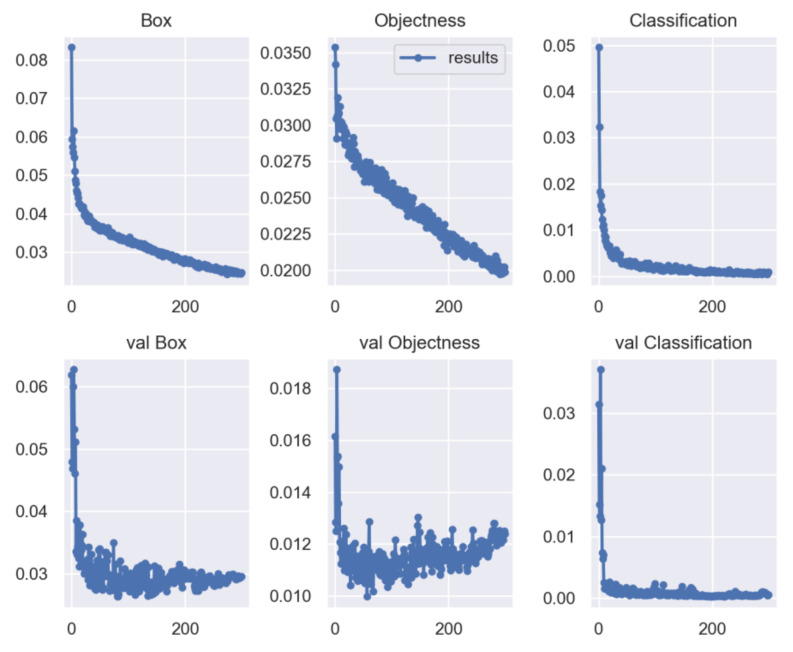
Boundary frame loss, location loss, and classification loss for the training and test results.

Figure 11 shows the training and test results for boundary frame loss, positioning loss, and classification loss. The box loss for target detection and location (box_loss) is approximately 0.03 for training and validation results, and boundary frame loss (obj_loss) is less than 0.02 for training and validation results. The result shows that objects and their positions can be correctly detected, albeit with some errors. The cls_loss display in both training and verification results is close to 0, in which case effective classification can be performed with little error.

**Figure 12:**
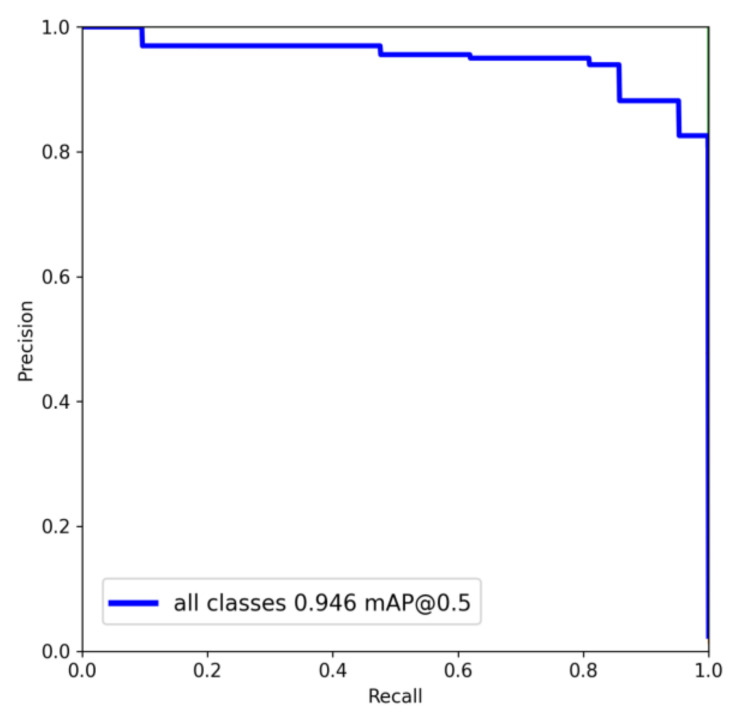
P-R curve.

Figure 12 shows the mapping relationship between model accuracy and defect detection of metal shaft recall during the training process. As seen from the figure, the total mAP value of this model can reach 94.6%. This value shows the capability of the trained model to accurately detect the surface defects on the metal shaft with high precision and recall value. The performance of the improved YOLOv5s model is shown in Table 1. Note that the improved algorithm achieves 90.7%, 95.3%, 94.6%, and 74.3%, respectively, for P, R, mAP@0.5, and mAP@0.5:0.95 of all categories. In addition, the recognition accuracy of scratches, pitted, and patches reaches 95.9%, 86.8%, and 95.1%. The experimental results show that the improved YOLOv5s network achieves high recognition accuracy for metal shaft defects.

**Figure 13:**
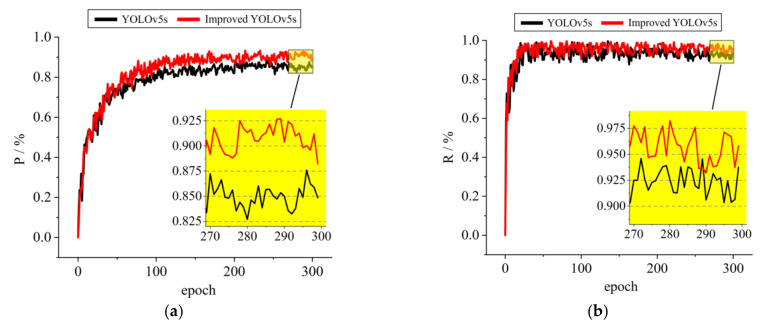

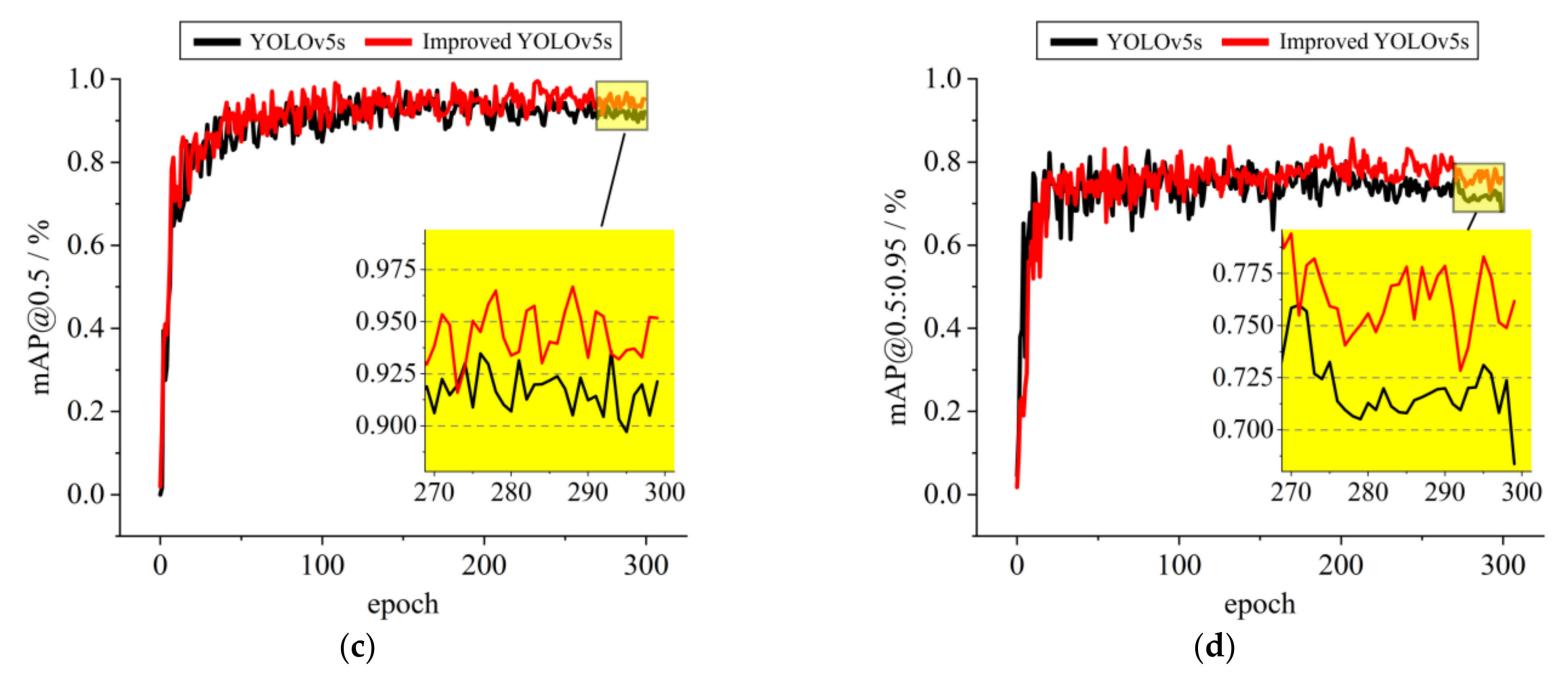
Accuracy change curve for YOLOv5s before and after improvement: (**a**) P change curve; (**b**) R change curve; (**c**) mAP@0.5 change curve; (**d**) mAP@0.5:0.95 change curve.

Figure 13 plots the accuracy change curves after 300 training iterations for the YOLOv5s model and the improved YOLOv5s model described in this paper. Among them, the black curve represents the change curve for the YOLOv5s model, and the red curve represents the change curve for the improved YOLOv5s model described in this paper. As can be seen from the large graphs, during the first 30 training iterations, the change curves of the three indexes for the YOLO5s model and the improved YOLOv5s model increase more and remain the same; during the training iterations after 30 times, the change curves of the three indexes for the two models gradually level off, and the P, R, mAP1, and mAP2 for the improved YOLOv5s model values are higher than those for the YOLOv5s model. As seen in the small graph after local enlargement, after the last 30 training iterations, the P, R, mAP@0.5, and mAP@0.5:0.95 for the improved YOLOv5s model described in this paper reach 90.7%, 95.3%, 94.6%, and 74.3%, respectively.

### 4.5. Ablation Experiments

To better verify the effectiveness of the improved algorithm to optimize the original algorithm, we conducted five sets of ablation experiments. Each set of experiments was validated using the same dataset of metal shaft defects for training. Table 2 shows the experimental results, with the optimal values in bold font.

The data in Table 2 show that after introducing the CBAM attention mechanism in the YOLOv5s backbone network, the mAP@0.5 improves by 3.1%, and the detection speed FPS decreases by 1.4 compared to the original YOLOv5s. After replacing the PANet in the YOLOv5s neck network with BiFPN, the mAP@0.5 improves by 1.0% compared to the original YOLOv5s, and the detection speed FPS decreases by 2.5. After using transfer learning, the mAP@0.5 improves by 2.5% compared to the usual training process, and the detection speed FPS reduces by 0.1. The experimental results show that each measure of improving YOLOv5s described in this paper improves performance compared to the original YOLOv5s network. The Precision, Recall, mAP@0.5, and mAP@0.5:0.95 improve by 5.1%, 2.8%, 3.5%, and 10.0%. Although the FPS is reduced by 2.8, it is within the controllable range, and the detection accuracy is greatly improved, which can realize the real-time detection of metal shaft defects.

### 4.6. Comparison with Other Networks

We take Precision, Recall, mAP@0.5, mAP@0.5:0.95, Memory Usage, and FPS as evaluation indexes to compare the improved YOLOv5s algorithm with other mainstream object detection algorithms. The performance of different networks on the dataset for detecting the metal shaft defects is shown in Table 3. Since the source code for YOLOXs does not count Precision and Recall, the table uses ‘--’ in the place of Precision and Recall for YOLOXs.

As shown in Table 3, the improved YOLOv5s algorithm is 7.3%, 8.9%, 6.0%, and 3.1% higher than Faster R-CNN, YOLOv3, SSD300, and YOLOv7 [40] at mAP@0.5, and 0.5% less than YOLOXs [41]. Regarding FPS, the improved YOLOv5s algorithm is 14.5, 7.3, 3.6, and 0.5 more than Faster R-CNN, YOLOv3, SSD300, and YOLOv7, respectively, and 1.6 less than YOLOXs. Although the improved YOLOv5s algorithm is slightly lower than YOLOXs in terms of detection accuracy, in terms of the Memory Usage index, the weight files used by the algorithm in this paper are smaller, which occupies less computer memory and attains a faster calculation speed. In addition, although YOLOv7 has the best detection effect on the COCO dataset with 80 categories, the detection effect could be better when trained on the self-made metal shaft defect dataset that is used in this paper.

### 4.7. Test Result

**Figure 14:**
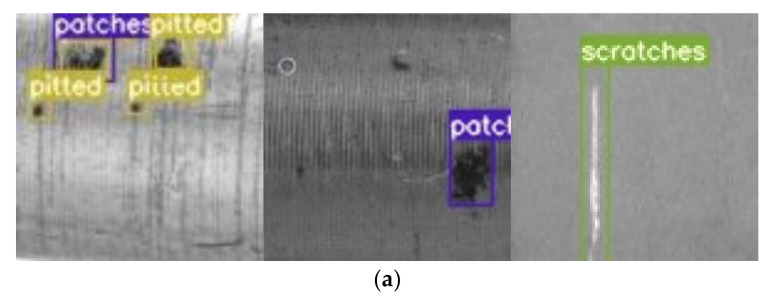

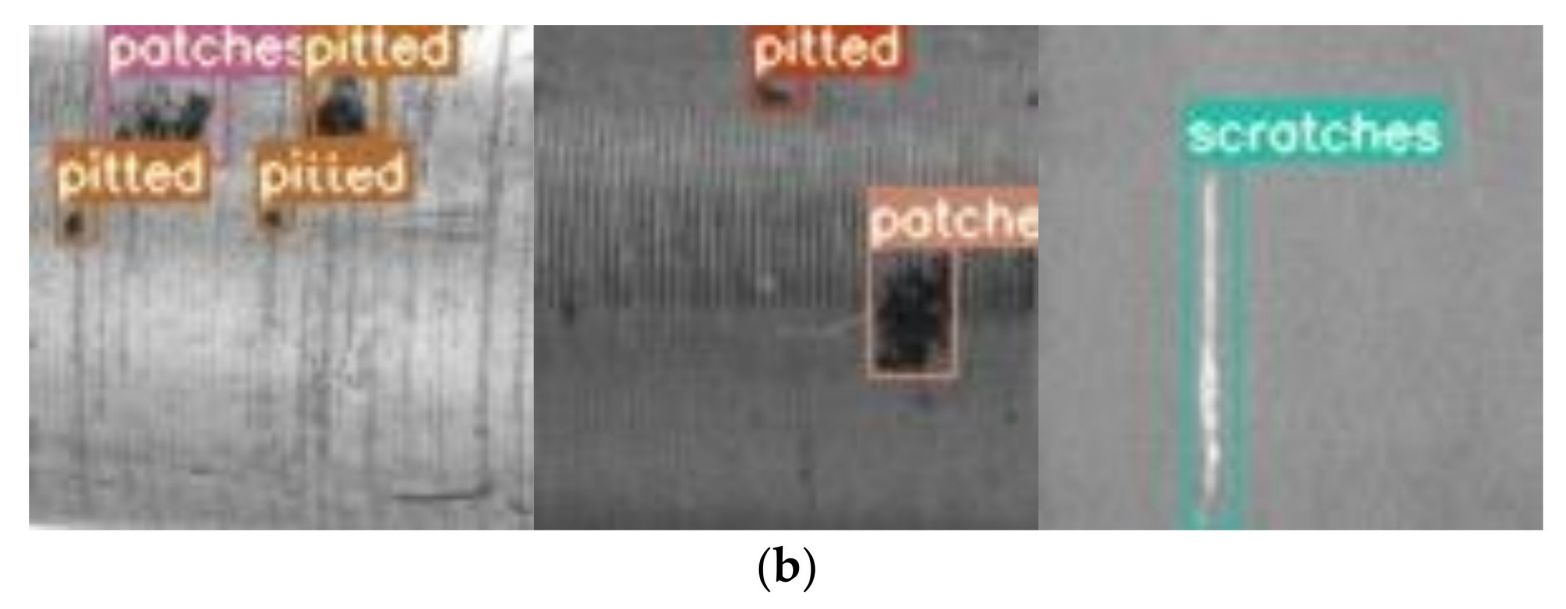
Test results of different surface defects: (**a**) defect detection effect of the original YOLOv5s; (**b**) defect detection effect of the improved YOLOv5s described in this paper.

To test and demonstrate the actual detection effect of the improved YOLOv5 network model described in this paper for metal shaft surface defects, we randomly selected pictures of different types of defects on the metal shaft surface. Figure 14a shows the defect detection effect of the original YOLOv5s, and Figure 14b shows the defect detection effect of the improved YOLOv5s described in this paper. The testing results prove that the algorithm used in this paper can detect defects quickly and accurately and returns accurate localization frames, proving the network’s effectiveness.

## 5. Conclusions

In this paper, we focus on solving the problem of insufficient accuracy for detecting defects on metal shaft surfaces. We add the CBAM attention module to the YOLOv5s model, which makes the network give more attention to the target regions containing important information, suppressing the influence of irrelevant information, and improving detection accuracy. We introduce the BiFPN module, which makes the network give more attention to feature mapping that contributes more to the output features and achieves better multi-scale feature fusion while realizing bi-directional feature information exchange. We use transfer learning to effectively avoid the problem of under-training the network due to the small number of actual defect data of the metal shaft surface and improve the model’s generalization ability.

The experimental results show that the improved YOLOv5s model has high detection accuracy for metal shaft surface defects. Additionally, compared with the original YOLOv5s model, the improved YOLOv5s model has higher detection accuracy with a 3.5% improvement in mAP, and the detection speed meets the requirements of actual defect detection, which verifies the effectiveness of the proposed algorithm. Finally, we trained and tested five mainstream detection models, Faster R-CNN, YOLOv3, SSD300, YOLOXs, and YOLOv7, and compared them with the improved algorithm described in this paper. We found that the improved model in this paper has higher detection accuracy. At the same time, the computing process occupies the least memory, which verifies the superiority of the algorithm. The improved YOLOv5 algorithm applied in this paper is effective and superior in detecting surface defects on metal shafts and provides a concrete theoretical basis for subsequent practical applications.

## Figures and Tables

**Table 1:** The performance of the improved YOLOv5s model.

Defects	Precision (%)	Recall (%)	mAP@0.5 (%)	mAP@0.5:0.95 (%)
scratches	90.9	98.4	95.9	80.2
pitted	89.6	86.2	86.8	66.1
patches	91.7	97.8	95.1	75.9
all	90.7	95.3	94.6	76.7

**Table 2:** Ablation experiments of the improved YOLOv5s.

Model	YOLOv5s	Improved YOLOv5s				
Add transfer learning	-	+	-	-	-	+
Add CBAM	-	-	+	-	+	+
Add BiFPN	-	-	-	+	+	+
Precision (%)	86.3	89.1	90.3	87.4	90.6	90.7
Recall (%)	92.7	93	94.1	93.8	95.1	95.3
mAP@0.5 (%)	91.4	93.7	94.2	92.3	94.5	94.6
mAP@0.5:0.95 (%)	72.1	69.4	70.7	68.2	72.5	76.7
FPS	19.5	19.4	18.1	17	17.4	16.7

**Table 3:** The performance of different networks for detecting defects in the metal shaft defect dataset.

Model	Precision (%)	Recall (%)	mAP@0.5 (%)	mAP@0.5:0.95 (%)	Memory Usage (MB)	FPS
Faster R-CNN	85.4	93.0	88.2	72.7	361	2.2
YOLOv3	87.8	84.1	86.9	70.6	237	9.4
SSD300	89.6	85.9	89.3	73.6	100	13.1
YOLOXs	--	--	95.1	78.4	68.7	18.3
YOLOv7	85.4	92.1	91.8	73.9	72.1	16.2
Improved YOLOv5s	90.7	95.3	94.6	74.3	14.1	16.7

## Data Availability

Not applicable.
